# The Various Faces of Creutzfeldt-Jakob Disease (CJD); a Case of CJD Presenting as Psychosis in a Middle-Aged Woman

**DOI:** 10.1192/bjo.2022.367

**Published:** 2022-06-20

**Authors:** Olusegun Sodiya, Akinkunmi Odutola, Michel Hakeem

**Affiliations:** Tees, Esk and Wear Valley NHS Foundation Trust, County Durham, United Kingdom

## Abstract

**Aims:**

Creutzfeldt-Jakob disease (CJD) is a rare, progressive, fatal neurodegenerative disorder caused by an abnormal glycoprotein known as the prion protein. The core features include progressive cognitive decline, cerebellar dysfunction, personality changes, and visual disturbances. Although psychiatric symptoms are rare, they can be the primary symptom of CJD, and such presentations can pose diagnostic difficulties. In this paper, we describe the case of Ms. R, who manifested psychotic symptoms as the first signs of CJD.

**Methods:**

Ms. R, was a 49-year-old white British female not previously known to psychiatric services, who presented with acute onset of florid psychotic symptoms. Her symptoms included auditory hallucinations, paranoia, and thought disorder. She was treated with antipsychotics for over four weeks, following her admission, but no improvement was seen. Instead, her psychosis worsened with cognitive decline, mutism, and the appearance of neurological symptoms such as jerky body movement, ataxia, and falls. All screening blood tests, chest X-ray, and CT abdomen were normal. The MRI, however revealed few patches of high T2/FLAIR signal in the deep white matter. Cerebrospinal fluid showed increased protein. Neurologist reviews suggested the possibility of sporadic CJD (sCJD) as a probable diagnosis. As her condition deteriorated, she became comatose and died four months after the appearance of the first psychiatric symptoms.

**Results:**

It can be challenging to diagnose CJD since the clinical picture overlaps with other neuropsychiatric and neurodegenerative conditions. It requires the presence of relevant clinical findings along with positive CSF, EEG, or MRI findings to make a probable diagnosis. Regarding our case, some noteworthy observations were psychosis as the initial symptom, relatively delayed onset of neurological signs, rapid deterioration with brief duration of illness. The MRI findings were typical of those seen in sCJD, although the EEG did not suggest sCJD. A differential of variant CJD was considered because of her age, prominent psychiatric symptoms, and delayed neurological signs.

**Conclusion:**

Creutzfeldt-Jakob disease course is rapidly progressive, and majority of patients die within one year. Therefore, awareness of early clinical features is of great significance. Among other things, this would enable patients and their families time to understand the nature of CJD, prognosis and prepare advanced directives. This case adds to the growing number of atypical presentation of CJD as well as pointing to an expanding spectrum of the disease. Therefore, clinicians should consider CJD in the differential diagnosis of new-onset psychosis, particularly if symptoms persist and worsen despite standard psychiatric treatment.

